# The Impact of Insulin Pump Therapy on Glycemic Regulation in Children and Adolescents with Type 1 Diabetes Mellitus—Preliminary Data from a Single Tertiary Pediatric Center

**DOI:** 10.3390/children13060819

**Published:** 2026-06-15

**Authors:** Maria Athanasopoulou, Maria Tsanti, Marios Papasotiriou, Alexandra Efthymiadou, Aristeidis Giannakopoulos, Dionisios Chrysis, Eirini Kostopoulou

**Affiliations:** 1 Division of Pediatric Endocrinology and Diabetes, Department of Pediatrics, University Hospital of Patras, University of Patras School of Medicine, 26504 Patras, Greece; athanasopoulou.maria01@gmail.com (M.A.); mariatsanti86@gmail.com (M.T.); eythymiadou@upatras.gr (A.E.); argianak@upatras.gr (A.G.); dchrysis@upatras.gr (D.C.); 2 Department of Nephrology and Kidney Transplantation, University Hospital of Patras, University of Patras, 26504 Patras, Greece; mpapasotir@upatras.gr

**Keywords:** type 1 diabetes mellitus (T1DM), continuous subcutaneous insulin infusion (CSII), automated insulin delivery (AID), insulin pump, glycemic regulation

## Abstract

**Highlights:**

**What are the main findings?**

- The transition of pediatric patients from multiple daily injections (MDI) to insulin pump therapy led to significant improvements in glycemic regulation (increased time in range, reduced time above range).
- In this exploratory analysis, automated insulin delivery (AID) technology demonstrated favorable improvements in specific sensor metrics compared to non-automated systems.

**What are the implications of the main findings?**

- Sensor-augmented technologies are more sensitive indicators of clinical improvement than traditional HbA1c.
- Improvement in the glycemic control of pediatric patients with T1DM after initiation of a pump therapy occurs without compromising patient safety.
- Awareness is raised among patients and their families regarding the benefits of AID systems.

**Abstract:**

**Background/Objectives:** Advanced technologies in type 1 diabetes mellitus (T1DM) management have reshaped the strategies used to achieve optimal glucose control. Continuous subcutaneous insulin infusion (CSII) and automated insulin delivery (AID) systems are effective alternatives to multiple daily injections (MDI). This study aims to evaluate glycemic regulation in children and adolescents transitioning from MDI to insulin pumps and to raise awareness among patients and their families regarding the benefits of these systems. **Methods:** 50 pediatric patients with T1DM (24 males, 26 females; mean age 10.76 ± 3.2 years) were evaluated. Cycle 1 established MDI metrics 3 months pre-transition. In cycle 2, patients transitioned either to an AID system (Medtronic MiniMed 780G, (Northridge, CA, USA), 78%), or a non-automated system (Omnipod DASH, 22%). Data were assessed at 3 and 6 months post-initiation. Parameters assessed were glycosylated hemoglobin (HbA1c), time in range (TIR), time above range (TAR), time below range (TBR), glucose management indicator (GMI) and coefficient of variation (CV). **Results:** The cohort exhibited a statistically significant increase in TIR (*p* = 0.0038) with mean values of 70.9% at 3 months and 73.2% at 6 months. TAR significantly reduced (*p* = 0.033) to 26.5% and 24.3% at 3 and 6 months, respectively. Sub-analysis in the AID group revealed a marked increase in TIR (*p* = 0.0001) alongside significant reductions in TAR (*p* = 0.0009) and GMI (*p* = 0.03). **Conclusions:** Transitioning from MDI to insulin pump therapy, particularly AID systems, leads to modest but significant improvements in specific sensor metrics (TIR, TAR) in real-world clinical practice. The consistency of these results across age groups indicates that AID systems can successfully overcome pediatric and adolescent diabetes management challenges.

## 1. Introduction

Type 1 diabetes mellitus (T1DM) is a chronic autoimmune disease characterized by the destruction of pancreatic beta cells within the islets of Langerhans [1]. It remains one of the most prevalent chronic conditions in childhood, although it may present at any age. Despite the rising incidence of type 2 diabetes mellitus (T2DM) among youth in recent years, T1DM remains the predominant form of diabetes in the pediatric population, accounting for over 90% of all diabetes cases in most Westernized countries [1,2,3,4].

Although the exact etiology of T1DM remains unclear, it is believed to result from interactions between genetic predisposition and environmental factors [1]. Proposed precipitating factors include maternal and intrauterine influences, viral infections, dietary factors and reduced early-life exposure to infectious agents, as suggested by the “hygiene hypothesis” [2,3,4]. Consequently, a process leading to the breakdown of immune self-tolerance is initiated, stimulating antigen-presenting cells to activate autoreactive T cells that progressively deplete pancreatic beta cells [2,3,4].

The incidence of T1DM has been increasing globally at an average annual rate of 3–4%. Many populations report a peak age of onset between 10 and 14 years [5]. According to data from the International Diabetes Federation (IDF), approximately 108,300 children and adolescents under 15 years of age were diagnosed with T1DM in 2021; a number that increased to 149,500 when extending the age range to individuals under 20 years [6]. Europe currently exhibits the highest incidence rates worldwide, with the IDF reporting 410,374 young people aged 0–19 years living with T1DM in 2024 [7]. In particular, the incidence among European children aged 0–14 years has doubled from 10.8 per 100,000 during 1994–2003 to 21.0 per 100,000 during 2013–2022. This corresponds to an estimated annual increase of 3.4% over 33 years [8]. Incidence increases with age, peaking between 10 and 14 years, consistent with global trends [6,9]. Regarding sex distribution, a higher incidence is generally observed in boys than in girls across most European countries [9]. Epidemiological trends in Greece mirror the broader European increase in T1DM incidence. Notably, a 25-year study conducted in Crete documented a 4.4% annual increase, particularly among children aged 5–14 years [10].

The rising global and European incidence of T1DM is influenced not only by genetic predisposition but also by multiple environmental and lifestyle-related factors. Childhood obesity is considered a significant contributor, potentially accelerating disease onset in genetically susceptible individuals [9]. Environmental influences such as those proposed by the hygiene hypothesis, early exposure to viral infections, and food-related chemicals have also been implicated [9]. Furthermore, seasonality in T1DM diagnosis has been observed, with incidence peaking during colder months [11]. This phenomenon is frequently attributed to the increased prevalence of seasonal viral infections and reduced exposure to ultraviolet radiation (UVR). Diminished UVB exposure may result in decreased vitamin D levels, a nutrient thought to exert a protective effect against T1DM [9]. Socioeconomic factors also appear to play an important role, as higher incidence rates are reported in countries with higher gross domestic product (GDP). This may reflect both improved screening practices and lifestyle-related environmental conditions prevalent in developed nations [9].

Following diagnosis and medical stabilization, an interprofessional healthcare team is responsible for providing diabetes education. This process is essential for the long-term management of this chronic condition, empowering both patients and their families to navigate the complexities of the disease effectively [12]. Core educational components include understanding insulin therapy, its purpose, mechanisms of action, delivery methods, injection site rotation, and dose adjustment. Education also focuses on blood glucose monitoring, including daily glucose targets and long-term glycemic control assessed through glycated hemoglobin (HbA1c). Additionally, patients and caregivers receive guidance on the effects of nutrition, exercise, and intercurrent illnesses on glycemic variability, as well as the prevention and management of acute complications such as hypoglycemia, hyperglycemia and ketosis [1,13,14,15].

Over the past two decades, the introduction of advanced technologies has transformed strategies for achieving optimal glycemic control. Continuous glucose monitoring (CGM), sensor-integrated insulin pump therapy and hybrid closed-loop systems are among these innovations. These technologies have promoted greater engagement of pediatric patients in self-management while also contributing to improved metabolic outcomes and quality of life [16,17].

Continuous subcutaneous insulin infusion (CSII), also known as insulin pump therapy, has been established as a safe and effective alternative to multiple daily injections (MDI) for over fifteen years [18,19]. Unlike conventional injection regimens, insulin pumps provide insulin delivery patterns that more closely resemble physiological basal insulin secretion and circadian fluctuations in insulin demand. Additionally, these devices allow the administration of micro-doses of insulin, which is particularly important in very young children, with infusion rates titratable to as low as 0.01 U/h [18,19,20]. Although evidence from the Diabetes Control and Complications Trial (DCCT) initially associated intensive insulin regimens with an elevated risk of hypoglycemia, more recent studies indicate that CSII is associated with lower rates of severe hypoglycemia—particularly nocturnal episodes—and diabetic ketoacidosis (DKA) compared to MDI therapy. CSII has also been linked to a reduced risk of long-term complications, including retinopathy and peripheral neuropathy [20]. Although CSII-related complications such as local infections and dermatological changes are relatively common, they are generally not associated with poorer glycemic control or discontinuation of insulin pump therapy [18,19,20].

Further technological advancements led to the development of sensor-augmented pump (SAP) therapy, which combines conventional insulin pump therapy with CGM systems. In SAP systems, glucose values are transmitted in real time to a dedicated reader, smartphone, or directly to the insulin pump interface. This provides users with continuous data, enabling proactive interventions such as administering correction boluses when predefined glucose thresholds are reached, rather than relying solely on intermittent fingerstick measurements. Although SAP therapy does not yet incorporate fully automated insulin dosing algorithms, it provides the essential data-driven framework upon which advanced automated systems are built [21].

In recent years, a major shift in diabetes technology has occurred with the emergence of automated insulin delivery (AID) systems, also referred to as hybrid closed-loop systems or “artificial pancreas” [18,22]. The initial development of this technology focused on sensor-augmented insulin pumps designed to reduce hypoglycemia. Several studies demonstrated that suspending basal insulin delivery in response to low sensor glucose effectively reduces hypoglycemic episodes [5,18,21,22]. Subsequent technological advancements enabled systems capable of suspending insulin delivery in anticipation of predicted hypoglycemia. However, the most significant breakthrough has been the development of “hybrid” closed-loop systems, which automatically adjust insulin delivery in response to both hyperglycemia and hypoglycemia. These systems integrate continuous glucose sensors with insulin pumps through controller algorithms that automatically modify insulin administration based on CGM data [18,20,21,22]. AID systems are associated with increased time in range (TIR), reduced time below range (TBR), improved HbA1c outcomes, enhanced metabolic control and improved quality of life in both pediatric and adult populations. Currently, all commercially available AID systems operate as single-hormone (insulin-only) systems. Dual-hormone AID systems incorporating glucagon or pramlintide, with the aim of more closely replicating physiological pancreatic function, are currently under development [18,21,22]. As of May 2026, two AID closed-loop systems are available in Greece -the Medtronic MiniMed 780G and the Eifron A8 TouchCare- alongside one CSII system, the Omnipod Dash. All systems, including CGM devices and necessary consumables, are fully reimbursed. This ensures no direct financial burden for families and facilitates access to diabetes technology, provided that patients are monitored at a certified pediatric diabetes center [23].

Although insulin pump therapy is beneficial for all children and adolescents with T1DM, certain age groups should be prioritized. Young children, particularly infants and toddlers aged 0–6 years, represent a high-priority group due to their heightened insulin sensitivity, minimal basal insulin requirements and unpredictable eating and activity patterns [24,25,26]. Consequently, minimizing hypoglycemia is a major therapeutic goal in this vulnerable population. Adolescents aged 12–18 years should also be prioritized due to challenges associated with the dawn phenomenon and the pronounced glycemic variability related to lifestyle changes [24]. Beyond age-specific considerations, several pediatric subgroups derive substantial benefit from these technologies in clinical practice, including patients with recurrent severe hypoglycemia, significant glycemic fluctuations despite acceptable HbA1c levels, needle phobia, adolescent pregnancy, competitive athletic participation or insulin regimens that compromise quality of life despite achieving target metabolic control [21].

Although AID and CSII systems currently represent the most advanced approaches in modern diabetes management, they still require manual user input for prandial boluses and physical activity adjustments. As these technologies become increasingly accessible, ongoing research focuses on reducing user–related barriers through the development of fully automated systems that may eventually eliminate the need for carbohydrate counting. By evaluating clinical outcomes three months prior to and at three and six months following technology initiation, this quality improvement project aims to determine how these theoretical advantages translate into real-world clinical improvements within a pediatric population. Specifically, this study seeks to (a) evaluate glycemic regulation in children and adolescents with T1DM following transition from intensified insulin therapy to insulin pump therapy, and (b) increase awareness among patients and their families regarding the benefits of insulin pump use, particularly in reducing complications and improving quality of life [21].

## 2. Materials and Methods

### 2.1. Study Criteria and Standards

The clinical efficacy of transitioning from MDI to insulin pump therapy was evaluated against international standards, established by the International Society for Pediatric and Adolescent Diabetes (ISPAD). Through the comparison between automated versus non-automated systems, these criteria were selected to assess whether the technological intervention successfully met the goals of improving glycemic stability and reducing hyperglycemic exposure [27,28]. The predefined criteria are presented in the following table (Table 1).

To successfully close the study loop, the primary measure of clinical efficacy is the achievement of statistical significance (*p* < 0.05) across the established metabolic markers. Specifically, the technological intervention is considered successful if a measurable and significant improvement is observed in HbA1c, TIR, TAR, CV and GMI when comparing the 3-month pre-intervention baseline against the 3 and 6-month post-initiation data. This statistical threshold ensures that the observed clinical improvements in glycemic control are consistent and attributable to the transition to insulin pump technology, rather than random variation.

To ensure clinical validity of the analyzed CGM metrics, strict criteria were applied at each evaluation period. In alignment with international consensus guidelines, only CGM datasets containing a minimum of 14 days of active sensor wear with an overall data completeness > 70% were eligible for analysis. This quality control measure guaranteed that the examined parameters (TIR, TAR, TBR, GMI, CV) accurately reflected the participants’ stable glycemic profiles throughout the assessment.

### 2.2. First Study Cycle (3 Months Prior to Intervention)

This study was conducted at the Pediatric Endocrinology Outpatient Clinic of the University General Hospital of Patras, Greece, a tertiary center with a 10-year experience in CSII use. The study aimed to evaluate the clinical impact of transitioning pediatric patients from MDI to either CSII or AID systems. All the patients who are currently in a CSII or AID system were included.

During the first study cycle, baseline clinical data were established to reflect the cohort’s glycemic status while on MDI therapy. Retrospective data were collected for the 3-month period prior to the intervention. The study cohort comprised a total of 50 pediatric patients with T1DM (24 males and 26 females) with a mean age of 10.8 ± 3.2 years. Twenty-six percent (26%, *n* = 13) were prepubertal. All participants had been on an intensified MDI regimen for at least 6 months prior to enrollment. During this 3–month baseline period, patients maintained active use of CGM devices to provide the necessary data for comparison against post-intervention metrics. The following data were assessed: HbA1c, TIR, TAR, TBR, GMI, and CV.

### 2.3. Intervention and Change Implementation

All 50 patients transitioned to insulin pump therapy and utilized CGM throughout the follow-up period. The cohort was stratified based on the selected device configuration as follows:

- In total, 39 patients (78%) transitioned to a fully AID system, the Medtronic MiniMed 780G (hybrid closed-loop system).
- In total, 11 patients (22%) transitioned to a non-automated system, the Omnipod DASH patch pump system.

Device selection (CSII or AID) was determined by the participants and their caregivers and all individuals were first-time pump users.

To ensure a smooth transition to pump therapy, comprehensive education was delivered through a team-based approach. Technical representatives from each manufacturer, accompanied by specialized diabetes nurse educators, conducted intensive training sessions for both the children and their families. This educational process was executed in close coordination with the center’s multidisciplinary medical team to standardize the implementation protocol and optimize initial system settings for each patient.

### 2.4. Second Study Cycle (3- and 6-Months Post Insulin Pump Initiation)

To close the study loop, follow-up data were collected at 3 and 6 months post insulin pump initiation (AID system or CSII). The core metabolic parameters (HbA1c, TIR, TAR, TBR, GMI, CV) were re-evaluated according to the Study criteria and standards (see Table 1). This follow-up phase served to determine whether the transition to insulin pump technology enabled the cohort to successfully achieve glycemic targets.

### 2.5. Statistical Analysis

Continuous variables are presented as means with standard deviations (SD). Statistical analyses were performed to identify significant changes across the three evaluation time points (the 3-month baseline and the sequential 3- and 6-month pump therapy intervals). For normality analysis of the continuous variables, the Shapiro–Wilk test was used. Data for measured parameters (HbA1c, TIR, TAR, TBR, GMI, CV) were analyzed using repeated measures ANOVA to compare mean values across the three time points, and mixed-effects models were used to account for missing case data points. The Tukey post hoc test was applied for multiple comparisons. All statistical analyses were carried out using either GraphPad Prism (version 8.0.2 for Windows, GraphPad Software, San Diego, CA, USA) or SPSS for Windows (version 16.0 SPSS Inc. Chicago, II, USA). All results were considered statistically significant at a threshold of *p* < 0.05.

The study was conducted in accordance with the Declaration of Helsinki and approved by the Research Ethics Committee of the University General Hospital of Patras. (Protocol number: 450, Date; 11 December 2025). Written informed parental consent was obtained for all participants before enrollment.

## 3. Results

During the first study cycle, the cohort of 50 pediatric patients (24 males and 26 females) was managed via an intensified MDI regimen. The mean diabetes duration of the study population was 3.76 ± 3.07 years for the AID group and 2.07 ± 1.55 years for the non-automated CSII group. To establish clinical homogeneity prior to the transition to insulin pumps, a comprehensive assessment of baseline CGM-derived parameters was performed during this initial study cycle, for each subgroup. Statistical analysis revealed no significant differences between the AID and the CSII subgroup. The results of the comparison of baseline CGM metrics between the two subgroups are represented in Table 2.

Overall, the metabolic profile of the entire cohort (N = 50) was characterized by a mean HbA1c value of 7.04%, a mean TIR of 67.50%, a mean TBR of 3.20% and a mean GMI of 6.97%. While these values indicate good glycemic control, a mean TAR value of 28.9% alongside a mean CV of 35.7% indicate frequent hyperglycemic episodes and borderline glucose variability (Table 3).

**Figure 1:**
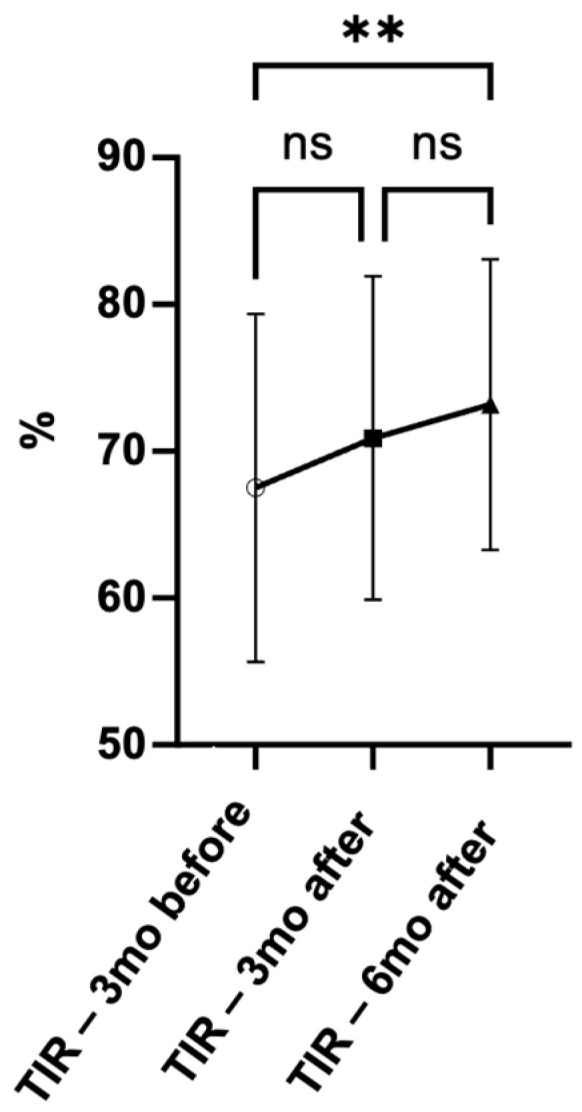
Evolution of TIR for the entire study cohort from baseline to 3 and 6 months post-intervention. **: *p* < 0.01.

**Figure 2:**
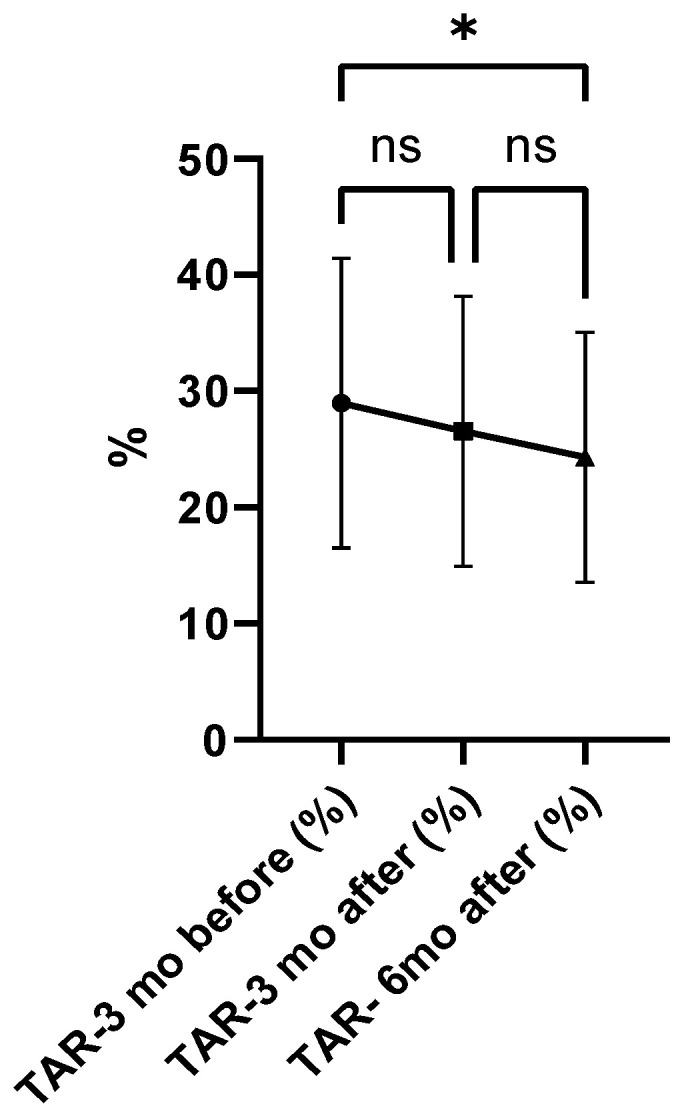
Evolution of TAR for the entire study cohort within both study cycles. *: *p* < 0.05.

The implementation of technological interventions through AID and CSII systems initiated the second study cycle. In total, 39 patients (78%) transitioned to a fully AID system, the Medtronic MiniMed 780G (hybrid closed-loop system), while 11 patients (22%) transitioned to a non-automated system, the Omnipod DASH patch pump system. Follow-up assessment at 3 and 6 months post-transition revealed a clear trend towards improved glycemic regulation. The total patient cohort exhibited a significant increase in TIR (*p* = 0.0038) rising to a mean value of 70.9% at 3 months and 73.2% at 6 months (Figure 1) and a reduction in TAR (*p* = 0.033) to 26.5% and 24.3% at 3 and 6 months, respectively (Figure 2). The above observations are presented in Table 3 and Table S1. No other glycemic parameters reached statistical significance when analyzing the cohort as a whole.

A critical safety endpoint of the study was the preservation of a low incidence of hypoglycemic episodes. TBR remained consistently low across all evaluation periods (mean value of 3.2% during the first cycle and of 2.5% for the 3- and 6-month assessments within the second cycle), staying well within the international safety TBR recommendations (<4%). This stability confirms that the observed improvements in TIR and TAR were achieved without increasing the risk of hypoglycemic events (Table 3).

**Figure 3:**
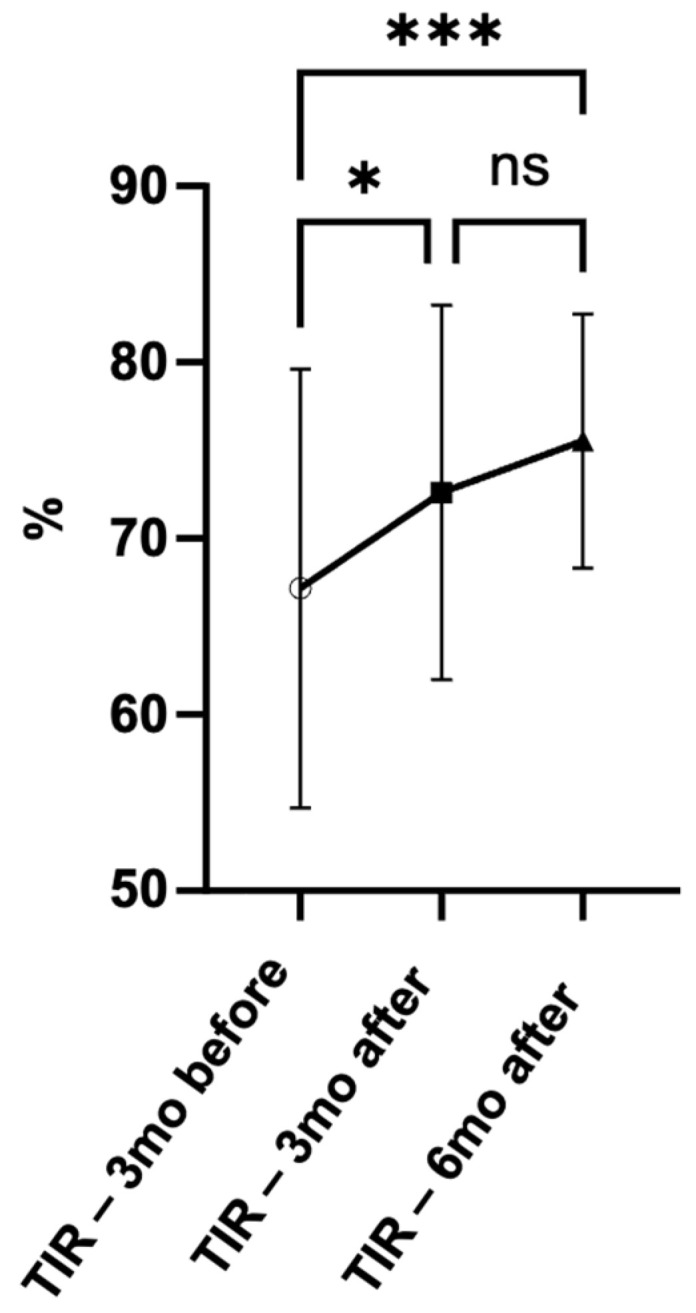
Assessment of TIR within the 2 study cycles within the AID subgroup. *: *p* < 0.05, ***: *p* < 0.001.

**Figure 4:**
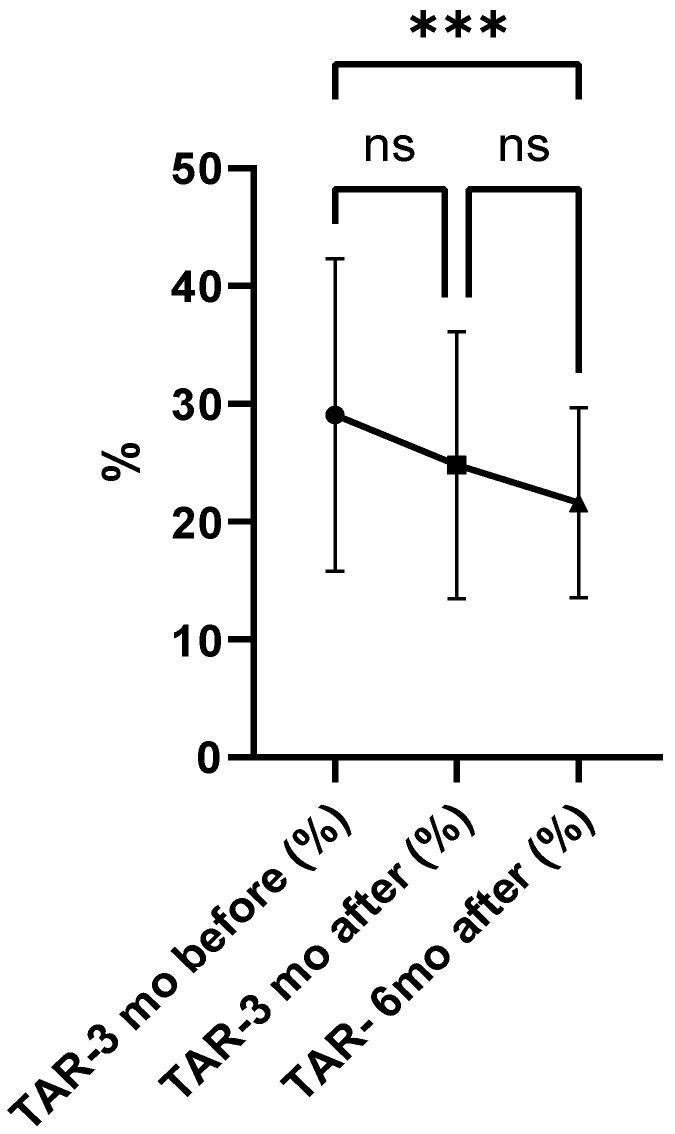
Evolution of TAR within both study cycles for the AID subgroup. ***: *p* < 0.001.

**Figure 5:**
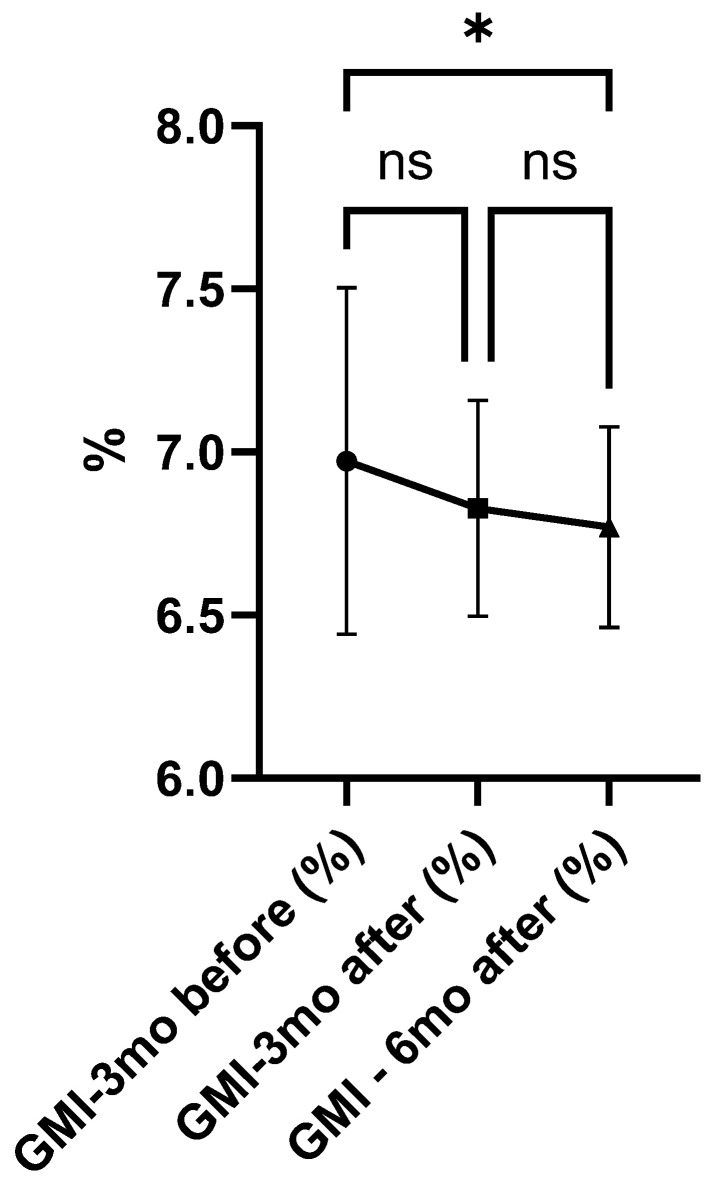
Changes in GMI for the AID group within the 2 study cycles. *: *p* < 0.05.

The comparative outcomes for key glycemic metrics across both study cycles are graphically illustrated in Figure 1, Figure 2, Figure 3, Figure 4 and Figure 5.

Subgroup analysis revealed that participants utilizing the Medtronic MiniMed 780G closed-loop system achieved a highly statistically significant increase in TIR (*p* = 0.0001; Figure 3) alongside significant reductions in both TAR (*p* = 0.0009; Figure 4) and GMI (*p* = 0.03; Figure 5). These observations are summarized in Table 4 and Table S2.

Additionally, patients utilizing the non-automated insulin delivery system demonstrated no statistically significant changes across any of the assessed metabolic markers. This suggests that the significant improvements in TIR and TAR within the entire cohort are likely associated with the automated system. However, selection bias regarding the choice of device remains an important factor.

The cohort was also stratified into two age groups: children < 10 years old and children ≥ 10 years old. The 10-year age threshold was used as a methodologically established marker that aligns with the clinical onset of puberty in the majority of pediatric cases. In general, puberty spans from 8 to 13 years in females and 9 to 14 years in males. While chronological age does not uniformly reflect biological puberty onset, this specific stratification remains widely accepted in pediatric endocrinology. It captures metabolic variations, hormonal fluctuations and growth-hormone-mediated insulin resistance associated with adolescence. The metabolic parameters for each defined age group are detailed in Table 5.

Furthermore, a statistically significant difference was observed in the proportion of patients who achieved the therapeutic target of TIR > 70% 6 months following initiation of pump therapy. In particular, 19 patients sustained the TIR target both pre- and post-transition. 3 patients reached the TIR target only pre-transition whereas 13 patients only post transition. Finally, 12 participants never achieved the TIR goal (McNemar test: *p* = 0.021). No significant differences were detected in the number of patients who achieved the HbA1c, TAR, TBR, GMI and CV goals both pre- and post-transition.

In addition, no episodes of severe hypoglycemia, DKA or device-related adverse events occurred during the study follow-up period.

## 4. Discussion

The findings of this clinical study demonstrate that transitioning pediatric patients from MDI therapy to insulin pump technology, particularly AID systems, led to significant improvements in glycemic regulation. Furthermore, within our cohort, the integration of controller algorithms for automated insulin delivery in AID technology was associated with more pronounced improvements in specific glycemic parameters over non-automated systems. This was evidenced by the reduction in glycemic variability and hyperglycemic excursions.

While the overall patient cohort demonstrated improvements in time in range (TIR) and time above range (TAR) values, the remaining glycemic parameters remained relatively stable. However, the Medtronic MiniMed 780G subgroup showed the most significant improvements in TIR, TAR and glucose management indicator (GMI), supporting the notion that automated insulin delivery aligns with enhanced glycemic outcomes. Due to the non-randomized, observational design of this sub-analysis, results should be interpreted with caution.

These outcomes are consistent with international evidence regarding the efficacy of AID and CSII systems. Pulkkinen et al. [29] reported an 8.3% increase in TIR alongside an 8.6% reduction in TAR over a 12-week period in children aged 2–6 years utilizing the MiniMed 780G system—findings that closely mirror the glycemic trends observed in our study. A significant reduction in HbA1c was also observed. Although the 18-month follow-up demonstrated sustained TIR improvement, the optimal threshold of 70% was not achieved, highlighting the challenges associated with glycemic management in younger children [29]. In contrast, our cohort, with a mean age of 10.76 years, surpassed this target, achieving a mean TIR of 73.2% at 6 months.

Similarly, Tornese et al. investigated the use of MiniMed 780G in children younger than 7 years and demonstrated a significant increase in TIR accompanied by a reduction in TAR—outcomes that closely align with our results [30]. Bombaci et al. evaluated the impact of AID systems at 15 days and 6 months post-initiation in children and adolescents previously managed with MDI regimens. Their findings demonstrated a sustained increase in TIR, further supporting the superiority of AID systems compared to MDI therapy [31]. Our findings regarding the MiniMed 780G subgroup are also in agreement with those reported by Collyns et al., who observed significant TIR improvement above the 70% target threshold in patients aged 7–14 years [32].

Regarding the Omnipod DASH subgroup, a study by Korkmaz et al. demonstrated overall improvement in metabolic control, even though HbA1c values remained above the recommended targets [33]. In addition, a meta-analysis by Martinez et al. highlighted the superiority of CSII over MDI therapy in achieving improved glycemic regulation, particularly through reductions in HbA1c and a substantially lower incidence of adverse events [34].

Although non-automated systems provide the technological foundation for integrated insulin delivery systems, their effectiveness largely depends on proactive patient engagement. Users remain responsible for dose calculations and basal insulin adjustments, leaving room for human error and delayed responses to glucose fluctuations [4,5].

Current clinical recommendations suggest that insulin pumps should be considered for all pediatric patients with T1DM through a collaborative decision-making process involving families and the multidisciplinary healthcare team [5,6]. The role of a multidisciplinary pediatric diabetes team is fundamental in providing continuous education, nutritional guidance and psychosocial support from the time of diagnosis, while also ensuring regular assessment of the transition from caregiver-led management to independent self-care [13,14].

Inadequate glycemic control may lead to acute complications, such as hypoglycemia, hyperglycemia and DKA, as well as long-term complications including cardiovascular disease, neuropathy, nephropathy, and retinopathy [1,14]. Therefore, frequent glucose monitoring is essential both during the day and overnight in order to minimize short- and long-term adverse outcomes. In pediatric and adolescent populations, diabetes management should also consider age-specific factors, including fluctuations in insulin sensitivity related to growth and pubertal development [13,14]. Furthermore, clinicians should evaluate each patient’s self-management capacity while remaining aware of neurological vulnerability of young children to hypoglycemia and hyperglycemia, as well as the potential neurocognitive consequences of DKA [13,14]. In our study, favorable outcomes were consistently observed across the entire cohort, whose mean age was 10.76 ± 3.2 years.

An important finding was the relative stability of laboratory-measured HbA1c despite the statistically significant improvements observed in TIR and GMI. Although HbA1c reflects average glucose levels over approximately three months, it fails to capture daily glucose variability. Since GMI is derived from real-time CGM data to estimate HbA1c, discrepancies between the two measurements may occur. Our findings suggest that sensor-augmented technologies may provide more sensitive indicators of clinical improvement than traditional HbA1c measurements. This observation is further supported by the study conducted by Korkmaz et al. [33], in which metabolic control improved despite HbA1c values remaining outside the normal range [33].

Another important finding was the maintenance of a low incidence of hypoglycemic episodes throughout the study. TBR remained consistently low, with mean values of 3.2% during the first cycle and 2.5% during the second cycle at both the 3- and 6-month assessments. These values remained well below the internationally recommended safety threshold of <4%, demonstrating the safety of the intervention. This safety profile was further underscored by the complete absence of any severe hypoglycemic episodes during the study. Our findings are consistent with those reported by Pulkkinen et al. [29], who observed no significant changes in TBR despite improvements in other glycemic parameters [29]. The safety profile observed in our study is further supported by the meta-analysis conducted by Martinez et al. [34], which demonstrated that transitioning to insulin pump therapy does not increase the risk of hypoglycemic events. Although the findings of the meta-analysis primarily reflect CSII devices, our study extends these observations to AID systems.

Within intensive insulin regimens, attempts to correct hyperglycemia often result in rebound hypoglycemia or increased risk of hypoglycemic episodes. In contrast, AID systems can automatically suspend insulin delivery as glucose levels approach the lower threshold, thereby enabling more effective glucose regulation. The successful implementation of these systems in our study may also be attributed to the close collaboration between technical representatives from manufacturing companies and the multidisciplinary healthcare team, who jointly provided continuous education, nutritional counseling, and psychosocial support.

Currently, the clinical efficacy of insulin pumps is well established through randomized controlled trials. Our study contributes valuable real-world evidence regarding the immediate effects of transitioning from MDI therapy to AID/CSII systems, thereby enriching the existing literature. To the best of our knowledge, this study provides some of the first real-world data regarding the clinical efficacy and safety of AID systems in a pediatric population in Greece. The short-term impact of these technologies was clearly demonstrated at both the 3- and 6-month follow-up assessments. In addition, while many studies focus on a single device, our research evaluated both automated and non-automated systems, thereby offering a broader perspective on diabetes management and glycemic regulation.

Despite these important findings, several limitations should be acknowledged. The relatively small sample size (*n* = 50) and the non-randomized allocation of insulin delivery systems may limit the generalizability of the results to broader pediatric populations. In particular, the observational and retrospective design of this study makes it susceptible to selection bias, as patients and their families autonomously selected their preferred insulin pump system. Consequently, although baseline HbA1c and diabetes duration were accounted for, our findings are likely affected by factors such as patient age, puberty stage, prior CGM use, daily insulin dose, BMI, family support, socioeconomic status, overall treatment engagement and clinician preference. Due to the retrospective nature of our data, these specific remaining variables could not be analyzed. Therefore, the differences observed between these groups cannot be solely attributed to the initiation of automated algorithms. These results should be interpreted as exploratory real-world data rather than definitive comparative evidence.

Furthermore, the comparative aspect of this study is limited by the small sample size of the non-automated CSII subgroup (*n* = 11) compared to the larger AID cohort. Our ability to detect statistical differences between the two groups is diminished, making this sub-analysis exploratory. Future larger-scale studies are required to confirm these real-world findings.

## 5. Conclusions

The findings of the present study suggest that transitioning from MDI therapy to insulin pump therapy—particularly AID systems—offers measurable benefits in T1DM management. These systems primarily reduce TAR and improve specific CGM metrics, such as TIR. However, these improvements did not translate into a statistically significant reduction in the entire cohort’s HbA1c and most secondary glycemic markers remained unchanged. Furthermore, the study highlights the safety of these technologies, as TBR remained consistently below the internationally recommended safety threshold of 4%, demonstrating that this glycemic control was achieved without compromising patient safety.

The consistency of these outcomes across a broad pediatric age range, although these real-world findings should be interpreted as exploratory, suggests that AID systems can effectively address many of the challenges associated with pediatric and adolescent diabetes management. Future research should focus on the long-term sustainability of these benefits, as well as the impact of these technologies on the quality of life of both children and their families.

## Figures and Tables

**Table 1:** Study criteria and standards.

Study Criteria	Clinical Standards (Targets)—ISPAD Guidelines	Rationale
Time in range (TIR)	Increase in TIR (Target: >70% of time within 70–180 mg/dL)	TIR is the primary metric for glycemic stability; improvements suggest successful insulin pump adjustment.
Time above range (TAR)	Reduction in TAR (Target: <25% of time above 180 mg/dL, or <5% of time above 250 mg/dL)	Reducing TAR is a key objective to prevent long-term complications.
Time below range (TBR)	Maintenance of TBR within safe limits (Target: <4% of time below 70 mg/dL or <1% of time below 54 mg/dL)	A core safety standard for insulin pump therapy is the reduction in hypoglycemic episodes.
Glycemic management indicator (GMI)	Reduction in GMI, particularly in automated system users (<7%).	GMI provides a sensor-derived estimate of HbA1c; reduction indicates improved overall metabolic control.
Coefficient of glycemic variation (CV)	Stabilization or reduction in CV (Target: ≤36%)	Lower glycemic variability indicates more predictable glucose patterns and a reduced risk of severe hypoglycemia.
HbA1c	Reduction in HbA1c (Target < 7.0%) or a statistically significant reduction from the 3-month pre-pump baseline.	HbA1c is the primary indicator of long-term glycemic control and a predictor of microvascular complication risk.

**Table 2:** Comparison of baseline CGM metrics between the AID and CSII subgroups.

Study Parameters	First Study Cycle—AID Group	First Study Cycle—CSII Group	*p*-Value (*t*-Test)
**TIR (%)**	67.17 ± 12.44	70.3 ± 9.82	0.485
**TAR (%)**	29.05 ± 13.25	35.58 ± 13.20	0.216
**TBR (%)**	3.22 ± 3.63	2.50 ± 3.42	0.597
**GMI (%)**	6.97 ± 0.53	6.96 ± 0.23	0.949
**CV (%)**	35.62 ± 5.86	36.04 ± 5.53	0.854
**HbA1c (%)**	6.98 ± 0.84	7.20 ± 0.78	0.499

All measurements are presented in mean ± SD.

**Table 3:** Mean values of examined study parameters for the whole cohort.

Study Parameters	First Study Cycle	3 Months Post Intervention	6 Months Post Intervention	*p*-Value (ANOVA)
TIR (%)	67.51 ± 11.85	70.33 ± 9.82	73.19 ± 9.89	0.0038
TAR (%)	28.96 ± 12.45	26.50 ± 11.61	24.30 ± 10.75	0.033
TBR (%)	3.23 ± 3.53	2.50 ± 2.67	2.55 ± 2.42	0.08
GMI (%)	6.97 ± 0.48	6.88 ± 0.35	6.83 ± 0.33	0.098
CV (%)	35.72 ± 5.74	34.31 ± 4.95	34.96 ± 3.84	0.12
HbA1c (%)	7.04 ± 0.82	6.95 ± 0.72	6.94 ± 0.74	0.74

All measurements are presented in mean ± SD.

**Table 4:** Mean values of examined study parameters for the AID group.

Study Parameters	First Study Cycle	3 Months Post Intervention	6 Months Post Intervention	*p*-Value (ANOVA)
TIR (%)	67.17 ± 12.44	72.61 ± 10.63	75.54 ± 7.21	0.0001
TAR (%)	29.05 ± 13.25	24.80 ± 11.31	21.62 ± 8.05	0.0009
TBR (%)	3.22 ± 3.63	2.45 ± 2.75	2.60 ± 2.47	0.14
GMI (%)	6.97 ± 0.53	6.83 ± 0.33	6.77 ± 0.30	0.03
CV (%)	35.62 ± 5.86	33.95 ± 4.43	34.56 ± 3.55	0.13
HbA1c (%)	6.98 ± 0.84	6.89 ± 0.73	6.92 ± 0.76	0.86

All measurements are presented in mean ± SD.

**Table 5:** Mean values of examined parameters according to age for the whole cohort.

**Age Group < 10 Years (*n* = 16)**				
**Study** **Parameters**	**First Study** **Cycle**	**3 Months Post Intervention**	**6 Months Post Intervention**	***p*** **-Value** **(ANOVA)**
TIR (%)	67.8 ± 12.6	70.7 ± 9.3	70.65 ± 13.23	0.31
TAR (%)	26.3 ± 13.9	25.3 ± 11	25.2 ± 14.8	0.81
TBR (%)	4.89 ± 4.7	3.61 ± 3.53	3.65 ± 3.16	0.19
GMI (%)	6.8 ± 0.54	6.85 ± 0.45	6.8 ± 0.48	0.84
CV (%)	37.5 ± 6.6	35.9 ± 4.3	35.8 ± 3.58	0.12
HbA1c (%)	6.97 ± 0.79	6.92 ± 0.68	6.66 ± 0.5	0.38
**Age group ≥ 10 years (*n* = 34)**				
TIR (%)	67.34 ± 11.6	70.99 ± 11.98	74.63 ± 7.26	0.008
TAR (%)	30.3 ± 11.63	27.2 ± 12	23.8 ± 7.83	0.02
TBR (%)	2.37 ± 2.41	1.91 ± 1.89	1.93 ± 1.64	0.34
GMI (%)	7.05 ± 0.43	6.9 ± 0.29	6.84 ± 0.23	0.17
CV (%)	34.8 ± 5.1	33.5 ± 5.13	34.5 ± 3.93	0.33
HbA1c (%)	7.07 ± 0.84	6.96 ± 0.75	7.07 ± 0.81	0.68

All measurements are presented in mean ± SD.

## Data Availability

The data that support the findings of this study are available from the corresponding author upon reasonable request.

## Supplementary Materials

The following supporting information can be downloaded at: https://www.mdpi.com/article/10.3390/children13060819/s1, Table S1. Multiple comparisons between different time points of examined parameters in the whole cohort of patients. Table S2. Multiple comparisons between different time points of examined parameters in patients treated with AID.

## Abbreviations

The following abbreviations are used in this manuscript:

T1DM	Type 1 diabetes mellitus
CSII	Continuous subcutaneous insulin infusion
AID	Automated insulin delivery
MDIs	Multiple daily injections
HbA1c	Glycosylated hemoglobin
TIR	Time in range
TAR	Time above range
TBR	Time below range
GMI	Glucose management indicator
CV	Coefficient of variation
T2DM	Type 2 diabetes mellitus
CGM	Continuous glucose monitoring
DCCT	Diabetes Control and Complications Trial
DKA	Diabetic ketoacidosis
SAP	Sensor augmented pump
ISPAD	International Society for Pediatric and Adolescent Diabetes
