# Supplementary material for: The Impact of Insulin Pump Therapy on Glycemic Regulation in Children and Adolescents with Type 1 Diabetes Mellitus—Preliminary Data from a Single Tertiary Pediatric Center
Source: Children (Basel). 2026 Jun 15;13(6):819. doi: 10.3390/children13060819 (PMC13297870; doi:10.3390/children13060819)
Supplement: Supplementary file 1 [file children-13-00819-s001.zip › children-4306210-supplementary.pdf]

Supplemental Table S1. Multiple comparisons between different time points of examined parameters in the whole cohort of patients.

Tukey's multiple comparisons test	Mean Diff,	95.00% CI of diff,	Adjusted *p* Value
	TIR		
First study cycle vs. 3 months post	−3.385	−7.230 to 0.4604	0.0948
First study cycle vs. 6 months post	−5.682	−9.657 to −1.707	0.0033
3 months post vs. 6 months post	−2.297	−5.213 to 0.6189	0.1479
	TAR		
First study cycle vs. 3 months post	2.403	−1.421 to 6.226	0.2916
First study cycle vs. 6 months post	4.664	0.6074 to 8.721	0.0208
3 months post vs. 6 months post	2.262	−0.8768 to 5.400	0.1996
	TBR		
First study cycle vs. 3 months post	0.7264	−0.2453 to 1.698	0.1783
First study cycle vs. 6 months post	0.6732	−0.4107 to 1.757	0.2984
3 months post vs. 6 months post	−0.05319	−0.5966 to 0.4902	0.9695
	GMI		
First study cycle vs. 3 months post	0.08581	−0.05731 to 0.2289	0.3242
First study cycle vs. 6 months post	0.1416	−0.01999 to 0.3031	0.0964
3 months post vs. 6 months post	0.05574	−0.02146 to 0.1329	0.1980
	CV		
First study cycle vs. 3 months post	1.401	−0.08623 to 2.888	0.0687
First study cycle vs. 6 months post	0.7521	−1.203 to 2.707	0.6230
3 months post vs. 6 months post	−0.6490	−2.059 to 0.7609	0.5096
	HbA1c		
First study cycle vs. 3 months post	0.09142	−0.1393 to 0.3221	0.6061
First study cycle vs. 6 months post	0.09635	−0.1478 to 0.3405	0.6067
3 months post vs. 6 months post	0.004924	−0.1585 to 0.1684	0.9970

Supplemental Table S2. Multiple comparisons between different time points of examined parameters in patients treated with AID.

Tukey's multiple comparisons test	Mean Diff,	95.00% CI of diff,	Adjusted *p* Value
	TIR		
First study cycle vs. 3 months post	−5.442	−10.02 to −0.8649	0.0166
First study cycle vs. 6 months post	−8.370	−12.81 to −3.928	0.0001
3 months post vs. 6 months post	−2.928	−6.250 to 0.3939	0.0931
	TAR		
First study cycle vs. 3 months post	4.246	−0.3046 to 8.797	0.0717
First study cycle vs. 6 months post	7.427	3.145 to 11.71	0.0004
3 months post vs. 6 months post	3.181	−0.3071 to 6.669	0.0798
	TBR		
First study cycle vs. 3 months post	0.7677	−0.3522 to 1.888	0.2306
First study cycle vs. 6 months post	0.6222	−0.6376 to 1.882	0.4584
3 months post vs. 6 months post	−0.1455	−0.7510 to 0.4601	0.8288

GMI			
First study cycle vs. 3 months post	0.1450	−0.02927 to 0.3192	0.1190
First study cycle vs. 6 months post	0.2029	0.01070 to 0.3951	0.0367
3 months post vs. 6 months post	0.05793	−0.03429 to 0.1502	0.2862
CV			
First study cycle vs. 3 months post	1.673	−0.1477 to 3.494	0.0771
First study cycle vs. 6 months post	1.058	−1.337 to 3.454	0.5318
3 months post vs. 6 months post	−0.6150	−2.331 to 1.102	0.6583
HbA1c			
First study cycle vs. 3 months post	0.09038	−0.2094 to 0.3902	0.7424
First study cycle vs. 6 months post	0.06130	−0.2423 to 0.3649	0.8737
3 months post vs. 6 months post	−0.02908	−0.2285 to 0.1704	0.9317
